# Effects of Axivity 3.0-based management model on pregnant women with gestational diabetes mellitus

**DOI:** 10.3389/fendo.2026.1722822

**Published:** 2026-02-12

**Authors:** Fengcheng Cai, Yingying Wu, Bei Liu, Mengyan Xu

**Affiliations:** 1 Department of Maternity Center, Hangzhou Women’s Hospital (Hangzhou Maternity and Child Health Care Hospital), Hangzhou, Zhejiang, China; 2 Department of Nutrition Center, Hangzhou Women’s Hospital (Hangzhou Maternity and Child Health Care Hospital), Hangzhou, Zhejiang, China; 3 Department of Nursing, Hangzhou Women’s Hospital (Hangzhou Maternity and Child Health Care Hospital), Hangzhou, Zhejiang, China

**Keywords:** delivery outcomes, exercise bracelet, exercise therapy, gestational diabetes mellitus, precision medicine

## Abstract

**Aims:**

To evaluate the effect of a precision exercise management model based on the Axivity 3.0 device on physical activity levels, glucose control, and delivery outcomes in patients with gestational diabetes mellitus (GDM).

**Patients and methods:**

This study employed a randomized controlled design. One hundred patients with GDM from Hangzhou Women’s Hospital from May 2020 to September 2020 who received regular obstetric follow-ups and intended to deliver in our hospital were recruited. Patients were randomly divided into a control group (conventional health education model of exercise during pregnancy) and a test group (received the Axivity 3.0-based management model combined with health education). A general information questionnaire, Pregnancy Exercise Self-efficacy Scale (P-ESES), Pregnancy Physical Activity Questionnaire; physical activity levels; fasting blood glucose (FBG), 2-hour postprandial blood glucose (2hPG); the duration of the first and second stages of labor for vaginally-delivering patients were assessed.

**Results:**

After the 4-week intervention, the total P-ESES score was significantly higher in the test group (median 39, IQR 36-42) than in the control group (median 36, IQR 33-38; p < 0.001). Weekly moderate-to-vigorous physical activity (MVPA) time increased to 75.95 ± 28.30 minutes in the test group versus 56.24 ± 23.66 minutes in the control group (mean difference: 19.71 minutes/week, p=0.001). At delivery, fasting blood glucose (FBG) and 2-hour postprandial glucose (2hPG) levels were significantly lower in the test group (FBG: 4.47 ± 0.47 mmol/L; 2hPG: 5.40 ± 0.56 mmol/L) compared to the control group (FBG: 4.82 ± 0.41 mmol/L, p=0.001; 2hPG: 6.02 ± 0.63 mmol/L, p<0.001). Total gestational weight gain was lower in the test group (11.95 ± 4.89 kg) than in the control group (14.32 ± 4.80 kg, p=0.030). Among vaginally-delivering patients, the median duration of the first and second stages of labor was shorter in the test group (389.5 and 44.5 minutes, respectively) than in the control group (510.0 and 51.0 minutes, respectively; p=0.015 and p=0.033).

**Conclusion:**

The Axivity 3.0-based precision management model was associated with significant improvements in exercise self-efficacy, increased moderate-to-vigorous physical activity, and better glycemic control and weight management in patients with GDM compared to conventional health education alone.

**Summary points:**

After the 4-week intervention, the test group showed a significantly higher total Pregnancy Exercise Self-Efficacy Scale (P-ESES) score (median 39 vs. 36 in controls, p < 0.001) and achieved greater weekly moderate-to-vigorous physical activity (MVPA) time (75.95 vs. 56.24 minutes, p=0.001). At delivery, fasting blood glucose (FBG: 4.47 vs. 4.82 mmol/L, p=0.001) and 2-hour postprandial glucose (2hPG: 5.40 vs. 6.02 mmol/L, p<0.001) levels were significantly lower in the test group compared to the control group. Total gestational weight gain was better controlled in the test group (11.95 kg) than in the control group (14.32 kg, p=0.030). Among vaginally-delivering patients, the median duration of the first stage (389.5 vs. 510.0 minutes, p=0.015) and the second stage (44.5 vs. 51.0 minutes, p=0.033) of labor was significantly shorter in the test group. The Axivity 3.0-based management model helps improve pregnancy exercise self-efficacy, elevate exercise time, and control blood glucose levels and weight gain in patients with GDM.

## 1 Introduction

Gestational diabetes mellitus (GDM) is a significant metabolic disorder that poses risks to both maternal and fetal health (1–3). For the mother, GDM is associated with an elevated risk of developing type 2 diabetes and cardiovascular complications later in life (4–6). For the infant, it can increase the likelihood of adverse outcomes such as polyhydramnios and respiratory distress (7–9). Management of GDM, particularly through lifestyle interventions like physical activity, is therefore crucial. However, sustaining adequate exercise during pregnancy is often hindered by motivational barriers, low self-efficacy, and a lack of objective monitoring and feedback. Wearable activity monitors present a promising tool to address these challenges by enabling precise tracking and personalized guidance. The Axivity device series, which includes the 3.0 model used in this study, is designed for accurate activity monitoring. This study aimed to evaluate a structured, precision management model utilizing the Axivity 3.0 device for women with GDM, with the goal of improving their physical activity levels, exercise self-efficacy, glycemic control, and pregnancy outcomes.

The current therapeutic approaches for patients with GDM include diet and exercise control, blood glucose monitoring, health education, maternal and infant monitoring, and medication. Among these, exercise treatment is a satisfactory alternative for patients with GDM without medical contraindications, helping to increase insulin sensitivity and reduce gestational insulin resistance, and exhibiting a promising result in the prevention of diabetes (10, 11). At present, exercise treatment usually relies on exercise intervention and exercise prescription, which lacks standard exercise management and makes it hard to perform a long-term follow-up (12). Additionally, it is difficult to obtain accurate data on exercise results, as the medical staff cannot acquire the real exercise level of pregnant women. This may contribute to a decrease in exercise willingness, limiting the curative effect of exercise treatment for patients with GDM.

Exercise self-efficacy, defined as an individual’s confidence in their ability to overcome barriers and persist with physical activity, is a key determinant of sustained behavior change (13, 14). The conception of the precision guidance model of exercise treatment for diabetes has been proposed in recent years (15). Bourgeoning literature verified the protective and effective role of exercise bracelet in blood glucose control by improving the enthusiasm and compliance of patients with diabetes (16–18). Until now, there are multiple wearable devices on the market, but their reliability in exercise intensity monitoring has not been well confirmed. Physical inactivity is a known modifiable risk factor for both GDM and future metabolic disorders (19). Lifestyle interventions that emphasize diet and exercise can markedly reduce the risk of progression to type 2 diabetes in women with a history of GDM (20). However, achieving sustained behavior change remains challenging, particularly due to poor self-efficacy and lack of real-time feedback. Wearable fitness devices offer a promising solution by providing objective, continuous monitoring of physical activity. Among them, Axivity 3.0 has emerged as a practical tool capable of tracking gait, exercise intensity, and duration in real time. By offering personalized feedback, it can support both clinical decision-making and patient motivation.

This study utilized the Axivity 3.0 exercise bracelet to guide exercise treatment for patients with GDM. We evaluated its effects on pregnancy exercise self-efficacy, blood glucose control, and maternal-infant delivery outcomes, providing a theoretical reference and a new management model for patients with GDM.

## 2 Materials and methods

### 2.1 Subjects

A total of 100 pregnant patients with GDM who received regular birth examinations and intended to give birth in Hangzhou Women’s Hospital from May 2020 to September 2020 were recruited. The diagnostic criteria for GDM followed the one-step guidelines established by the International Association of Diabetes and Pregnancy Study Groups (IADPSG) in 2010 (21). The patients with GDM were divided into two groups: the control group (n=50) and the test group (n=50).

### 2.2 Sample size calculation and randomization

Sample size calculation was conducted using a power analysis with an alpha level of 0.05 and a power of 0.80. Based on previous studies, we anticipated a mean difference of 30 minutes per week in moderate-to-vigorous physical activity (MVPA) and a difference of 0.5 mmol/L in 2-hour postprandial glucose (2hPG) between the two groups. With an estimated standard deviation of 40 minutes for MVPA and 0.8 mmol/L for 2hPG, a minimum of 40 participants per group was determined to be sufficient for statistical power. To account for potential dropouts, we aimed to recruit 50 participants per group. Randomization was performed using a computer-generated random number sequence prepared by an independent statistician not involved in recruitment or intervention delivery. The allocation sequence was concealed using sequentially numbered, opaque, sealed envelopes. Due to the nature of the intervention (device vs. no device), blinding of participants and intervention staff was not feasible. However, outcome assessors and data analysts were blinded to group allocation where possible.

### 2.3 Inclusion and exclusion criteria

The inclusion criteria were shown as follows: 1) Primipara, fetal cephalic position, singleton pregnancy, 24–28 weeks gestation, age between 20–34 years old; 2) GDM was diagnosed at 24–28 weeks of gestation by oral glucose tolerance test (OGTT); 3) No mental and cognitive dysfunction, the patient can effectively communicate with researchers; 4) Low level of physical activity (moderate intensity exercise) before or during pregnancy [moderate-to-vigorous intensity physical activity (MVPA) < 150 min (21), monitored by an Axivity 3.0 exercise bracelet]. The exclusion criteria were listed as follows: 1) Communication difficulty; 2) Advanced maternal age (age > 35 years); 3) Uteroplacental blood flow reduction diseases during pregnancy such as pregnancy-induced hypertension syndrome, placenta previa, multiple pregnancies, polyhydramnios, inevitable abortion and threatened premature labor; 4) Abnormal fetal position including breech position, transverse position, etc. 5) Patients with a history of smoking, abnormal heart function, asthma, bone and joint disease, pubic separation and abnormal body structure. Dropout standards include: 1) Loss to follow-up; 2) Unstable blood glucose control patients requiring medicine; 3) Patients who dropped out of the intervention study; 4) Delivery in other hospitals.

During the study period, participants whose blood glucose control became unstable and required initiation of medication (such as insulin or metformin) were considered to have met the dropout criterion and were excluded from the final analysis. No participants in either group required medication during the intervention period. The observed reductions in fasting and postprandial glucose levels are therefore attributable to lifestyle interventions rather than pharmacological treatment.

### 2.4 Intervention strategy

The patients in the control group were given a one-day clinic health education course on GDM, and the patients in the test group received the Axivity 3.0-based management model, based on the health education of GDM. Additionally, both groups participated in weekly follow-up visits to discuss physical activity, nutrition, and overall pregnancy care (22). The key difference between the groups was that the test group received the Axivity 3.0-based management model, which included individualized feedback based on the physical activity data collected from the bracelet. The one-day educational course includes 1) The definition of GDM and its detrimental impact on the mother and infant. 2) The importance of nutritional therapy on blood glucose levels and nutritional requirements for patients with GDM. 3) Food calorie calculation and the application of food exchange methods. 4) Diet provided by the Nutrition department, such as breakfast ratio, including a boiled egg 50g, 1.5 pieces of whole wheat toast 35g, dried bean curd mixed with celery 40g, food exchange number of about 3, the total calorie is 270 kcal. 5) The types and benefits of exercise during pregnancy. 6) Pregnancy exercise safety, moderate-intensity exercise demonstration, patient and medical staff exercise together for 20 minutes. 7) Blood glucose monitoring methods and precautions during pregnancy, including emergency treatment for hypoglycemia. 8) Diagnosis and treatment consultation from nutrition doctors, the information will be filed by the nutrition department, and the patient will be followed up.

As for patients with GDM in the test group, the Axivity 3.0 exercise bracelet-guided precision management model was implemented based on the one-day clinic health education, the detailed procedure is outlined as follows: 1) Conduct appropriate medical screenings for patients with GDM (23), the exercise contraindications and criteria for exercise termination were informed in detail to ensure the safety of the subjects. 2) Inform the precautions for wearing Axivity 3.0 according to the instructions, and the indicators such as intensity and duration of physical activity. 3) Collect activity data weekly by retrieving the bracelets. Data included daily physical activity duration and intensity, particularly MVPA time. 4) Establish individual records and provide personalized feedback. If MVPA time was below the recommended 150 min/w, medical staff would analyze the reason for insufficient activity based on both Axivity data and patient exercise diaries. 5) Use the Borg Rate of Perceived Exertion (RPE) (24) and the 2011 Compendium of Physical Activities (CPA) (25) to assess and adjust exercise prescriptions. For example, a patient with consistently low MVPA might be encouraged to substitute sedentary behaviors with brisk walking (3.5 METs) for 30 minutes per day. 6) Ongoing monitoring and adjustment of MVPA goals were implemented weekly, based on updated bracelet data and patient feedback. 7) Communication among patients to share the experience of exercise during pregnancy.

Axivity 3.0 exercise bracelet and three scales including the general information questionnaire, the Pregnancy Physical Activity Questionnaire (PPAQ) and Pregnancy Exercise Self-efficacy Scale (P-ESES) were applied here.

### 2.5 General information questionnaire

The study was designed and established by postgraduates, obstetricians, nutritionists, and experts in the hospital according to the published literature. The contents were listed as follows: 1) General information: name, health number, age, education level, height, weight, body mass index (BMI), residence during pregnancy, occupation; 2) Obstetric data: gestational number, gestational weeks, pregnancy complications, OGTT results, glycated hemoglobin (HbAlc) results; 3) Exercise data: pre-pregnancy exercise habits, recognition of the benefits of exercise for pregnancy, exercise preferences, and duration of moderate physical activity each week.

### 2.6 Pregnancy physical activity questionnaire

PPAQ, established by American scholars (26), is utilized to assess the 1-week physical activity energy consumption for pregnant women, or the time and frequency of each activity type. PPAQ has been widely applied worldwide, such as in Vietnam, Japan, Australia, etc., and it has good reliability and validity in various cultural backgrounds. The PPAQ included 33 physical activities, and there were 31 physical activities from 4 categories (8 exercise activities, 14 housework activities, 5 work activities and 4 transportation activities) in this study after excluding some activity items that are not common in China such as weeding. The Chinese version of the PPAQ was translated by Zhang et al. (27), with a content validity of 0.940 and a retest reliability of 0.944, and the correlation coefficient between the questionnaire and the energy consumption recorder was 0.768, indicating that the translation version of PPAQ is suitable for the investigation of physical activity during pregnancy in China. According to the analysis method of PPAQ, the amount of physical activity time per week could be calculated, including the amount of time spent in physical activity, as well as light, moderate and high activity intensity.

### 2.7 Pregnancy exercise self-efficacy scale

Revised P-ESES contains three aspects, which are overcoming emotional disorders, overcoming exercise disorders, and overcoming support disorders (28). There are a total of 10 items, all items used the 5-grade Likert scoring method, including strongly disagree, disagree, neutral, agree and strongly agree. The corresponding scores are 1–5 points, and the total range is 10–50 points. The higher score indicated a higher sense of exercise self-efficacy. The self-exercise efficacy was divided into three levels according to the total score, including low (10-20), moderate (21-40) and high (41-50). The Chinese version of P-ESES was translated by Yang et al. (29), and the Crobach’s coefficients were 0.730, 0.705 and 0.669, respectively, ensuring the good internal consistency, reliability and validity of the Chinese version of P-ESES in Chinese pregnant women.

### 2.8 Axivity 3.0 exercise bracelet

The Axivity 3.0 exercise bracelet features a three-axis acceleration sensor that collects acceleration changes during human body activity, intelligently identifies the physical activity, and accurately calculates corresponding activity mode, activity intensity and amount. Rowlands et al. (30) confirmed that the Axivity 3.0 exercise bracelet reliably measures physical activity intensity. The Axivity 3.0 Exercise Bracelet has been used in large-scale epidemiological investigations by the British Biobank, demonstrating its effectiveness and reliability in monitoring exercise conditions (31). The Axivity 3.0 exercise bracelets used in the current study were provided by Westlake University. For this research, the devices were provided at no cost to the participants. It is acknowledged that the typical market price for the Axivity 3.0 or similar research-grade activity monitors at the time of the study was approximately USD $200–300 per unit, which is a consideration for broader clinical implementation.

### 2.9 Evaluation index

This study primarily evaluates blood glucose levels, specifically FBG and 2hPG. FBG and 2hPG were measured at enrollment, after 4 weeks of intervention, and at delivery. For each time point, blood glucose values represent the mean of two consecutive measurements taken within one week to enhance reliability. Other evaluation indexes include 1) Exercise self-efficacy and PPAQ questionnaire of patients with GDM four weeks after the intervention; 2) The time amount of weekly moderate-intensity activity/exercise after the intervention; 3) Perinatal outcomes of mothers and children in the two groups after delivery, for instance, delivery mode, duration of labor, postpartum hemorrhage, neonatal weight, neonatal asphyxia, neonatal hypoglycemia, gestational hypertension, premature delivery and the condition of gestational weight gain.

### 2.10 Statistical analysis

Data processing and statistical analysis were conducted using SPSS 22.0 software. Two individuals recorded the data to minimize potential errors. Normality of continuous variables was assessed using the Shapiro-Wilk test and visual inspection of Q-Q plots. Measurement data were represented as mean ± standard deviation (SD) when they conformed to the normal distribution. If the data did not follow a normal distribution, they were reported as median (M) and interquartile range (IQR) and analyzed using non-parametric tests (Mann-Whitney U test for between-group comparisons, Wilcoxon signed-rank test for within-group comparisons). For between-group comparisons of continuous outcomes at single time points (e.g., post-intervention), independent samples t-tests (parametric) or Mann-Whitney U tests (non-parametric) were used as appropriate. To adjust for baseline values and potential confounders (pre-pregnancy BMI, baseline MVPA, education level), analysis of covariance (ANCOVA) was performed for key continuous outcomes (e.g., post-intervention MVPA time, FBG, 2hPG), with baseline scores as covariates. For within-group comparisons (pre vs. post), paired t-tests or Wilcoxon signed-rank tests were used. To address multiple comparisons, the Bonferroni correction was applied when comparing multiple related outcomes within a family (e.g., the three subscales of P-ESES), adjusting the significance level accordingly. For other exploratory comparisons, uncorrected p-values are reported with a note of caution regarding interpretation. Enumeration data were presented as frequency and proportion (%), with the chi-square test or Fisher’s exact test used for comparisons between groups. For statistically significant results (*p* < 0.05), 95% confidence intervals (CIs) and effect sizes (Cohen’s d for parametric tests, r for non-parametric tests) are reported alongside *p*-values to provide estimates of precision and magnitude. *p* < 0.05 was considered statistically significant for primary outcomes.

### 2.11 Covariate adjustment

To account for potential confounding factors, we included pre-pregnancy BMI, educational level, and baseline moderate-to-vigorous physical activity (MVPA) as covariates in the analysis of primary outcomes (blood glucose levels, physical activity time, and P-ESES scores) using analysis of covariance (ANCOVA). Dietary intake was assessed at baseline using a 24-hour dietary recall, and the total daily calorie intake was calculated. However, due to the lack of continuous dietary monitoring throughout the intervention period, dietary data were not included as a covariate in the final adjusted models. Instead, we acknowledged the potential influence of dietary changes as a limitation in the discussion section.

## 3 Results

### 3.1 General information

**Figure 1:**
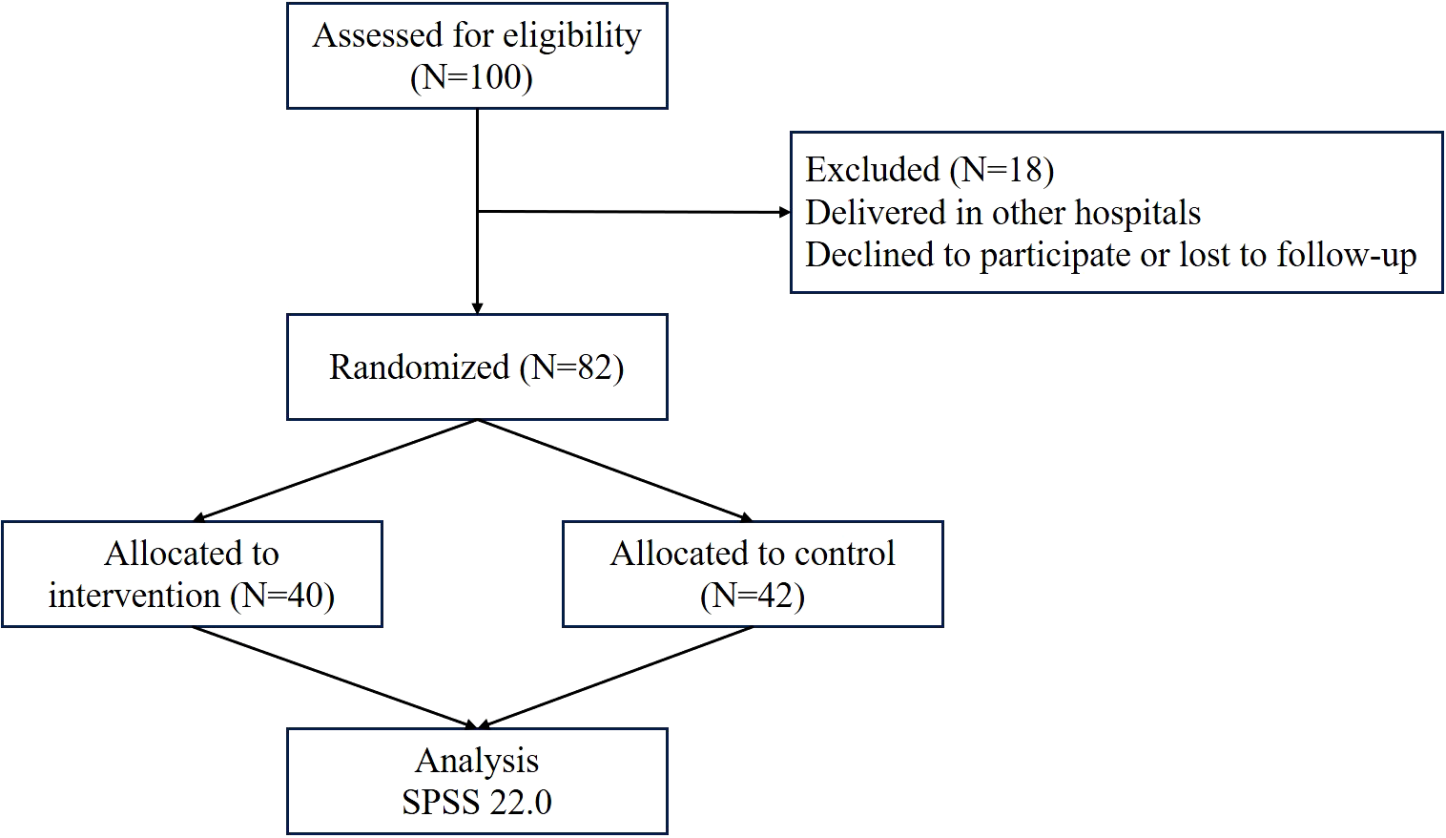
CONSORT CONSORT flow diagram of participant enrollment and allocation. Of the 100 pregnant women with GDM assessed for eligibility, 82 were successfully enrolled and randomized into the study (test group: n=40; control group: n=42). The remaining 18 participants were excluded prior to randomization due to delivering in other hospitals or declining to participate/lost to follow-up. This accounts for the discrepancy between the initial assessment number (N = 100) and the randomized number (N = 82).

This study enrolled 100 patients with GDM. After excluding those who withdrew, were lost to follow-up, or delivered elsewhere, 82 patients remained for analysis—40 in the test group and 42 in the control group (**Figure 1**). As shown in **Table 1**, the average age of pregnant women with GDM in the two groups was 29 years old (range: 23-34), and the gestational weeks were 24–28 weeks. There were no significant differences in indexes in general information and pre-pregnancy exercise between the two groups of patients with GDM (*p>*0.05) (**Tables 1**, **2**). Baseline dietary intake was also assessed via 24-hour recall, but as it was not included as a covariate in the primary analysis, detailed dietary data are not presented here.

**Table 1:** General information of patients with GDM in the two groups.

Index	Test group (n=40)	Control group (n=42)	*X^2^/t/z*	*P*
Age (year)	29.90 ± 2.60	29.52 ± 2.62	0.659	0.512
Height	160(158-164)	160.5(158-163)	0.140	0.889
Pre-pregnancy weight	57.16 ± 8.53	54.70 ± 6.81	1.448	0.152
Body weight at enrollment	64.34 ± 8.72	63.94 ± 8.49	0.211	0.833
Pre-pregnancy BMI	22.08 ± 2.92	21.10 ± 2.33	1.689	0.095
Residence (%)			0.176	0.675
Urban	34(85.0)	38(90.5)		
Rural area	6(15.0)	4(9.5)		
Educational level (%)			2.692	0.611
Junior high school and below	1(2.5)	1(2.4)		
High school or technical secondary school	3(7.5)	1(2.4)		
Junior college	10(25.0)	7(16.7)		
Regular college	22(55.0)	26(61.9)		
Master and above	4(10.0)	7(16.7)		
Gravidity (n)			0.734	0.392
1	24(60.0)	29(69.0)		
≥2	16(40.0)	13(31.0)		
Abnormal OGTT (%)			1.07	0.785
Fasting	12	14		
1-hour postprandial	8	8		
2-hour postprandial	11	14		
≥2 items	9	6		
HbAlc%	4.66 ± 0.55	4.68 ± 0.48	0.107	0.915
Gestational weeks at enrollment	25(24.5-26)	25(24-26)	0.316	0.752
Gestational weeks at delivery	38.5(38-39)	38(38-39)	0.129	0.898

**Table 2:** Exercise-related data of patients with GDM in the two groups before the intervention.

Index	Test group (n=40)	Control group (n=42)	*X^2^/t/z*	*P*
Attitude to exercise (%)			1.774	0.412
Like	10(25)	8(19.0)		
Dislike	3(7.5)	7(16.7)		
Neutral	27(67.5)	27(64.3)		
Exercise is good for health (%)			0.464	0.496
Helpful	40(100)	40(95.2)		
Useless	0(0)	0(0)		
Uncertain	0(0)	2(4.8)		
Frequency of physical activity before pregnancy (%)			3.09	0.543
Occasional	13(32.5)	19(45.2)		
Sometimes	15(37.5)	13(31)		
Often	8(20.0)	4(9.5)		
Once a day	3(7.5)	5(11.9)		
Several times a day	1(2.5)	1(2.4)		
Exercise time before pregnancy (%)			5.148	0.161
<5 minutes	2(5.0)	1(2.4)		
5 ~15 minutes	5(12.5)	9(21.4)		
16 ~30 minutes	25(62.5)	17(40.5)		
>30 minutes	8(20.0)	15(35.7)		
Frequency of physical activity after pregnancy (%)			8.339	0.080
Occasional	6(15.0)	8(19)		
Sometimes	8(20.0)	13(31)		
Often	16(40.0)	6(14.3)		
Once a day	5(12.5)	11(26.2)		
Several times a day	5(12.5)	4(9.5)		
Exercise time after pregnancy (%)			4.546	0.208
<5 minutes	2(5)	0(0)		
5 ~15 minutes	9(22.5)	7(16.7)		
16 ~30 minutes	22(55.0)	30(71.4)		
>30 minutes	7(17.5)	5(11.9)		
MVPA time in one week before enrollment (min)	42(18, 71.5)	41(27, 89)	0.715	0.475

### 3.2 Comparison of blood glucose indexes of patients with GDM in the two groups before and after intervention

Key findings are summarized in the **Table 3**. At baseline, there were no significant differences in fasting blood glucose (FBG) or 2-hour postprandial glucose (2hPG) levels between the test and control groups (both *p* > 0.05). Following the 4-week intervention, both groups showed a reduction in blood glucose levels. However, while there was no significant between-group difference in FBG at this time point (*p* > 0.05), the 2hPG level was significantly lower in the test group compared to the control group (*p* = 0.016). At delivery, patients in the test group demonstrated significantly lower levels of both FBG and 2hPG compared to those in the control group (FBG: 4.47 ± 0.47 vs. 4.82 ± 0.41 mmol/L, *p* = 0.001; 2hPG: 5.40 ± 0.56 vs. 6.02 ± 0.63 mmol/L, *p* < 0.001). Within-group comparisons revealed a continuous, significant decline in both FBG and 2hPG from baseline to delivery in the test group (all *p* < 0.05). In contrast, the control group showed an initial reduction at 4 weeks but failed to maintain this improvement, with no significant further decline in glucose levels by the time of delivery (*p* > 0.05).

**Table 3:** Comparison of blood glucose indices (FBG and 2hPG) in patients with GDM before and after intervention.

Group	n	Enrollment (mean ± SD)	4-week post-intervention (mean ± SD) [95% CI]	Delivery (mean ± SD) [95% CI]	*P*-value (time)	*P*-value (group)	Effect size (d)
FBG (mmol/L)							
Test	40	5.51 ± 0.27	4.80 ± 0.52* [4.63, 4.97]	4.47 ± 0.47*# [4.32, 4.62]	<0.001	0.002	0.75
Control	42	5.57 ± 0.34	4.92 ± 0.41* [4.79, 5.05]	4.82 ± 0.41* [4.69, 4.95]	<0.001		
2hPG (mmol/L)							
Test	40	7.45 ± 0.66	5.87 ± 0.76* [5.63, 6.11]	5.40 ± 0.56*# [5.22, 5.58]	<0.001	0.001	0.82
Control	42	7.33 ± 0.59	6.29 ± 0.80* [6.04, 6.54]	6.02 ± 0.63* [5.82, 6.22]	<0.001		

*p < 0.05 vs. Enrollment within group; #*p* < 0.05 vs. 4-week post-intervention within group. *P*-values for time and group effects from repeated measures ANCOVA. Effect size (Cohen’s d) calculated for between-group difference at delivery.

### 3.3 Comparison of P-ESES scores of patients with GDM in the two groups before and after intervention

As detailed in the consolidated **Table 4**, baseline P-ESES total and subscale scores did not differ significantly between groups (all *p* > 0.05). After the 4-week intervention, the test group showed a significant increase in the total P-ESES score compared to both its own baseline (*p* = 0.001) and the control group post-intervention (*p* < 0.001; effect size r = 0.42). This improvement was consistent across all three subscales: overcoming exercise disorders (*p* = 0.007 within group, *p* < 0.001 between groups, r = 0.46), overcoming emotional disorders (*p* < 0.001 within group, *p* < 0.001 between groups, r = 0.42), and overcoming support disorders (*p* = 0.003 within group, *p* = 0.009 between groups, r = 0.28). In contrast, the control group exhibited no significant change in either the total P-ESES score or any of its subscales from baseline to post-intervention (all *p* > 0.05).

**Table 4:** Comparison of pregnancy exercise self-efficacy scale total and subscale scores in patients with GDM before and after intervention.

P-ESES measure	Group	n	Before intervention median (IQR)	After intervention median (IQR)	Within-group *p*-value a	Between-group *p*-value b (Post-intervention)	Effect Size (r) c
Total Score	Test	40	35 (33 – 38)	39 (36 – 42)*	0.001	<0.001	0.42
	Control	42	37 (32 – 38)	36 (33 – 38)	0.267		
Subscale: Overcoming Exercise Disorders	Test	40	15.5 (14 – 16)	16.0 (15.0 – 17.5)*	0.007	<0.001	0.46
	Control	42	15.5 (14 – 16)	15.0 (14 – 16)	0.089		
Subscale: Overcoming Emotional Disorders	Test	40	7.0 (6 – 7)	8.0 (7 – 8)*	<0.001	<0.001	0.42
	Control	42	6.0 (6 – 8)	7.0 (6 – 7)	0.071		
Subscale: Overcoming Support Disorders	Test	40	14.0 (13 – 15)	15.0 (14 – 16)*	0.003	0.009	0.28
	Control	42	15.0 (13 – 16)	15.0 (13 – 15)	0.088		

GDM, Gestational Diabetes Mellitus; P-ESES, Pregnancy Exercise Self-Efficacy Scale; IQR, Interquartile Range. A Within-group *p*-values were derived from the Wilcoxon signed-rank test, comparing scores before and after the intervention within each group. b Between-group p-values were derived from the Mann-Whitney U test, comparing post-intervention scores between the test and control groups. Baseline comparisons showed no significant differences (all *p* > 0.05, data not shown in table for brevity). c Effect size (r) was calculated for the between-group difference in post-intervention scores. An r of 0.1, 0.3, and 0.5 is considered a small, medium, and large effect, respectively. * indicates the comparison after intervention, the test group showed significant improvements in these indicators.

### 3.4 Comparison of exercise level of patients with GDM in the two groups before and after intervention

The results of PPAQ questionnaire showed that the amount of exercise time and housework time in GDM test group increased after the intervention, along with improvements in MVPA and total exercise time. Further comparison results between groups indicated that there was no statistical significance in MVPA scores between the two groups at baseline. After a 4-week intervention, the test group achieved an average of 75.95 minutes/week of MVPA, compared to 56.24 minutes/week in the control group (mean difference: 19.71 minutes/week, *p* = 0.001). Additionally, sedentary time decreased by 20.40 minutes/week in the test group versus no significant change in the control group (*p* = 0.011). Similar statistically significant improvements were observed in moderate-intensity time, housework, occupational activities, and exercise time (all *p* < 0.05, **Table 5**). To track the progression of exercise engagement, we compared weekly moderate-to-vigorous physical activity (MVPA) time across multiple time points (**Table 6**). Inter-group comparisons revealed no statistically significant difference in baseline MVPA between the two groups, confirming their comparability. Within the test group, MVPA time showed a significant and progressive increase from baseline to the 4th, 8th, and 12th weeks post-intervention (all pairwise comparisons, p < 0.05). In the control group, MVPA time at the 4th week was significantly higher than baseline but plateaued thereafter, with no significant differences among the 4th, 8th, and 12th-week time points. Between-group comparisons at each post-intervention time point (4th, 8th, and 12th weeks) revealed that the test group consistently achieved significantly higher weekly MVPA times than the control group (all p < 0.001, **Table 6**).

**Table 5:** Comparison of PPAQ of patients with GDM in the two groups before and after intervention (MET-h/week).

Item	Group	Case	Before intervention	After intervention	*t/z*	*p*
Total time of physical activity	Test	40	119.36 ± 35.68	136.99 ± 36.61	2.323	0.025
	Control	42	119.91 ± 38.49	124.12 ± 38.71	1.814	0.077
	*t*		0.067	1.546		
	*p*		0.947	0.126		
Sedentary time	Test	40	80.72 ± 30.54	60.32 ± 30.62	3.142	0.003
	Control	42	77.2 ± 29.28	77.74 ± 29.72	0.364	0.718
	*t*		0.534	2.613		
	*p*		0.595	0.011		
Moderate-intensity exercise time	Test	40	2.4(2.04, 2.4)	3.6(3.01, 5.98)	4.799	<0.001
	Control	42	2.4(1.68, 2.4)	2.4(1.95, 3.6)	1.246	0.213
	*z*		0.367	3.310		
	*p*		0.713	0.001		
High-intensity exercise time	Test	40	0(0, 0)	0(0, 0)	1.604	0.109
	Control	42	0(0, 0)	0(0, 0)	0.000	1.000
	*z*		0.000	1.797		
	*p*		1.000	0.072		
Housework	Test	40	13.13(8.4, 28.7)	46.15(38.33, 60.2)	4.938	<0.001
	Control	42	12.43(8.05, 28.18)	15.14(8.05, 30.28)	2.814	0.005
	*z*		0.804	5.644		
	*p*		0.421	0.000		
Occupational activity	Test	40	35.53(33.25, 71.05)	33.25(22.05, 52.85)	2.310	0.021
	Control	42	52.85(33.25, 75.25)	52.85(17.85, 71.05)	1.597	0.110
	*z*		1.179	2.275		
	*p*		0.238	0.023		
Exercise	Test	40	2.4(2.04, 2.71)	4.55(3.21, 6)	4.604	<0.001
	Control	42	2.4(1.68, 2.4)	2.4(1.95, 3.55)	1.246	0.213
	*z*		0.826	4.081		
	*p*		0.409	0.000		

**Table 6:** Comparison of weekly moderate-to-vigorous physical activity time (minutes) across study time points in the two groups.

Group	n	Before intervention	Four weeks after intervention	Eighth week after intervention	Twelfth week after intervention
Test	40	47.58 ± 14.63	75.95 ± 28.30^*^	129.31 ± 36.16^*#^	144.1 ± 29.60^*#△^
Control	42	47.64 ± 19.42	56.24 ± 23.66^*^	58.57 ± 27.48^*^	59.31 ± 23.60^*^
*t*		-0.018	3.428	9.956	13.406
*p*		0.986	0.001	<0.001	<0.001

*indicates the comparison after intervention, p<0.05; ^#^indicates the comparison before intervention, p<0.05; ^△^ indicates the comparison before intervention, p<0.05.

The inter-group comparison results showed no statistically significant difference between the two groups before intervention, indicating the comparability between the groups. The comparison results before and after intervention in the test group showed that the pairwise comparison differences between two time points were statistically significant. As the intervention time prolonged, statistically significant differences after intervention and before intervention were observed in the control group, but there was no statistically significant difference in the time points of the fourth, eighth, and twelfth weeks after intervention. The differences between the two groups at the fourth, eighth, and twelfth weeks after intervention were statistically significant, with the test group being higher than the control group.

### 3.5 Delivery outcomes of patients with GDM in two groups

To assess the protective role of Axivity 3.0 exercise bracelet on patients with GDM, delivery outcomes in two groups were analyzed. The findings indicated that weight gain control was more effective in the test group than in the control group (*p* < 0.05), but no significant differences were found in other indicators between the two groups (**Table 7**). Additionally, the results showed that the labor duration for vaginal delivery was significantly shorter for patients with GDM in the test group during stages 1 and 2 compared to the control group (median stage 1: 389.5 min vs. 510.0 min, p=0.015; median stage 2: 44.5 min vs. 51.0 min, p=0.033; **Table 8**). There were no significant differences in neonatal blood glucose-related indicators were found between the two groups (**Table 9**).

**Table 7:** Delivery outcomes of patients with GDM in two groups.

Index	Test group (n=40)	Control group (n=42)	*X^2^/z*	*p*
Weight gain after intervention	4.92 ± 3.28	8.04 ± 7.12	2.551	0.013
Total weight gain during pregnancy	11.95 ± 4.89	14.32 ± 4.80	2.215	0.030
Hypertensive diseases during pregnancy			0.003	0.955
Yes	3(7.5)	2(4.8)		
No	37(92.5)	40(95.2)		
Delivery mode (%)			0.36	0.549
Vaginal delivery	30(75.0)	29(69.0)		
Caesarean section	10(25.0)	13(31.0)		
Postpartum hemorrhage (%)			0.236	0.627
Yes	4(10.0)	2(4.8)		
No	36(90.0)	40(95.2)		
Neonatal asphyxia (%)			0.000	1.000
Yes	1(2.5)	2(4.8)		
No	39(97.5)	40(95.2)		
Fetal macrosomia (%)			0.131	0.717
Yes	2(5.0)	4(9.5)		
No	38(95.0)	38(90.5)		
Premature delivery (%)			0.003	0.955
Yes	3(7.5)	2(4.8)		
No	37(92.5)	40(95.2)		

**Table 8:** Comparison of labor duration (min) of vaginal delivery in two groups of patients with GDM.

Item	Test group (n=30)	Control (n=29)	*z*	*p*
Stage 1	389.5(280-485)	510.0(365-635)	2.441	0.015
Stage 2	44.5(36-51)	51.0(44-62)	2.131	0.033
Stage 3	6.5(5-12)	10.0(7-14)	1.814	0.070
Total stage of labor	447.0(350-535)	565.0(425-680)	2.699	0.007

**Table 9:** Comparison of neonatal blood glucose (mmol/L) in two groups of patients with GDM.

Index	Test group (n=40)	Control group (n=42)	*X^2^/z/t*	*P*
Neonatal blood glucose (1)	3.2(2.6-3.7)	3.15(2.5-4.1)	0.009	0.993
Neonatal blood glucose (2) (%)			0.903	0.342
<2.6	7(17.5)	11(26.2)		
≥2.6	33 (82.5)	31 (73.8)		
Neonatal blood glucose (3) (%)			2.352	0.125
<2.2	3(7.5)	8 (19.0.)		
≥2.2	37 (92.5)	34 (81.0)		
Oral sugar water (%)			3.527	0.060
Yes	11(27.5)	20(47.6)		
No	29(72.5)	22(52.4)		
Transfer to NICU (%)			0.000	1.000
Yes	3 (7.5)	3(7.1)		
No	37(92.5)	39(92.9)		

## 4 Discussion

Appropriate physical activity during pregnancy plays a vital role in promoting perinatal health and reducing the risk of adverse outcomes such as gestational diabetes mellitus (GDM). To effectively prevent and manage GDM, it is essential to adopt a practical and efficient model for monitoring key exercise indicators (32). In this study, we applied the Axivity 3.0 exercise bracelet within a precision-guided management framework for patients with GDM. Our findings demonstrated that this model was associated with improvements in exercise levels and pregnancy-related exercise self-efficacy, as well as better blood glucose control and some maternal outcomes. These results suggest that the Axivity 3.0-based management approach may hold promise in supporting the development of a healthier lifestyle among women with GDM. However, given the relatively short intervention period and the observational nature of some associations, causal inferences should be made cautiously.

The observed link between improved self-efficacy, increased physical activity, and better glucose control is consistent with social cognitive theory and is correlational in the context of this study design. Our findings align with recent studies utilizing other wearable devices (e.g., Fitbit, ActiGraph) in gestational diabetes management, which also report improvements in physical activity adherence and glycemic parameters when combined with behavioral support. However, our study adds specificity by utilizing the Axivity device, which has been validated in large cohorts like the UK Biobank, and by implementing a structured precision management model with weekly feedback.

It is recommended that physical activity during pregnancy is set as MVPA with a certain duration and frequency (33). However, physical activity during pregnancy remains at a low level worldwide, therefore, it is an urgent requirement to develop an effective management model. The precision management model refers to the scientific management system that maximizes the hit rate and accuracy (34, 35). In this study, the Axivity3.0 exercise bracelet used in this study could monitor exercise intensity level, which is one of the modern scientific monitoring tools. Our results verified that Axivity3.0 wearing could gradually increase the frequency and time amount of MVPA. The quantified MVPA time of patients in the test group was 45.63 min/week before intervention and then increased to 75.9 min/w after a 4-week intervention. With the delivery looming, although some pregnant women elevated their amount of exercise to 144.1 min/w, this level still fell short of the 150 min/w target set by this study’s intervention protocol. Intriguingly, the MVPA time still significantly increased in the test group after the intervention. With the assistance of Axivity 3.0 exercise bracelet, precise quantification and feedback of MVPA time amount can be obtained, which helps our patients comprehend the present exercise information and strengthen self-management.

Our further analysis confirmed that the Axivity 3.0 exercise bracelet provides beneficial precision guidance for women with GDM, facilitating a transition from sedentary lifestyles to more active routines such as housework. Previous studies have indicated that pregnant women typically engage in a single type of exercise, such as climbing stairs or walking (36, 37), which aligns with our baseline findings. Following the intervention involving the Axivity 3.0 exercise bracelet, pregnant women who predominantly walked for exercise shifted towards moderate walking mode and began incorporating other forms of physical activity, such as upper limb exercise and elastic band use. The updated exercises are consistent with exercise guidelines recommended in Europe and America, including muscle strength training or resistance exercise (32). Self-efficacy theory, proposed by Bandura, is termed as the confidence a person has about their capacity to undertake healthy behavior. Specifically, exercise self-efficacy refers to people’s belief that they can successfully overcome various difficulties and insist on exercise (38), which is one of the most vital influencing factors for pregnant women to keep exercise behavior. Notably, we noticed that the total P-ESES scores in both test and control groups were at a moderate efficacy level, furthermore, our results indicated that after intervention with Axivity 3.0 exercise bracelet, the total score of P-ESES in the test group was higher than that in the control group, as well as the improved three aspects of self-efficacy during pregnancy. These findings are consistent with previous studies (39, 40), demonstrating that higher exercise self-efficacy could further stimulate the self-exercise motivation of pregnant women.

Blood glucose is the golden indicator to evaluate the efficiency of the management model in patients with GDM. According to the Chinese GDM diagnostic guidelines published in 2014, the recommended blood glucose control standards during pregnancy are FBG ≤ 5.3 mmol/L, and 2hPG ≤ 6.7 mmol/L (41). In this study, the blood glucose indexes including FBG and 2hPG of patients with GDM in both test and control groups were declined, and the FBG and 2hPG control were better in the test group. We speculated that a one-day clinic health education course is beneficial for patients with GDM to promote their exercise in both groups (42), while the pregnant women in the control group lacked effective exercise supervision and management as time went by, and their increased abdominal size and discomfort of physical activity limited their exercise insistency. Long-term appropriate exercise during pregnancy could induce the alternation of skeletal muscle fiber, thereby improving glucolipid metabolism (43, 44). Besides, glucose uptake by muscles and internal organs is compromised in women with GDM due to reduced insulin sensitivity (45). Previous studies have proved that exercise could improve insulin sensitivity and insulin-induced muscle glucose uptake in type 2 diabetes, hence we hypothesized that exercise may also elicit similar beneficial biological effects for patients with GDM.

In this study, we further explored the delivery outcomes of patients with GDM treated by the Axivity 3.0-based management model. Generally, the pre-pregnancy BMI for normal-weight women during pregnancy ranges from 18.5 to 24.9 kg/m^2^, with an appropriate weight gain range of 11.5–16 kg, averaging at 12.5 kg. Here, the gestational weight gain of patients with GDM in the test group was significantly lower than that in the control group. Previous literature demonstrated that unhealthy lifestyles, including sedentary and insufficient physical activity, are important factors contributing to overweight during pregnancy (46, 47). Our findings align with recent studies utilizing other wearable devices (e.g., Fitbit, ActiGraph) in gestational diabetes management, which also report improvements in physical activity adherence and glycemic parameters when combined with behavioral support (48–50). The Axivity 3.0 exercise bracelet utilized in this study offers a precise guidance and management mode, which can effectively urge and remind pregnant women to exercise and increase the amount of MVPA, and then reach the goal of weight gain control during pregnancy. Our results further verified the Axivity 3.0 exercise bracelet-based model reduced the duration of labor for vaginal deliveries in patients with GDM during stages 1 and 2. A stable mood and free position preference after labor brought by regular exercise may help shorten the stages of labor.

This study has several limitations. First, subjects were recruited from a single, large-scale specialized hospital in a provincial capital, which may limit the generalizability of our findings to other settings, such as rural areas or different healthcare systems. Second, while we provided standardized dietary education, we did not intensively monitor or control dietary intake throughout the study period. Dietary changes could have confounded the observed effects on blood glucose and weight. Additionally, data from the exercise bracelet required retrieval with specific equipment, meaning that exercise feedback was not provided in real time, which may reduce immediate motivational impact compared to real-time feedback devices. Fourth, the sample size, though calculated *a priori*, was modest, which may have limited our power to detect significant differences in some secondary outcomes, particularly neonatal indicators. While the more recent Axivity 6.0 is available, the Axivity 3.0 device still has a large user base, and its core activity monitoring functionality remains consistent. Finally, no significant differences in neonatal blood glucose-related indicators were observed; future studies with larger sample sizes and longer follow-up are needed to investigate effects on neonatal outcomes.

## 5 Conclusion

In this study, the Axivity 3.0-based management model was associated with improvements in exercise self-efficacy, physical activity levels, and glycemic control in patients with GDM compared to standard care. This model appears particularly suitable for patients with GDM who receive regular obstetric follow-up and are capable of using digital devices. These findings suggest it may be considered for integration into outpatient health education and primary care systems to support the development of healthier lifestyles and potentially improve maternal and neonatal outcomes.

## Trial registration

The study was registered in the Chinese Clinical Trial Registry (registration number: ChiCTR2400082811).

## Data Availability

The original contributions presented in the study are included in the article/supplementary material. Further inquiries can be directed to the corresponding author.
